# Aboveground carbon of community-managed Chirpine (*Pinus roxburghii* Sarg.) forests of Nepal based on stand types and geographic aspects

**DOI:** 10.7717/peerj.6494

**Published:** 2019-03-08

**Authors:** Shiva Pariyar, Liubov Volkova, Ram P. Sharma, Ramesh Sunam, Christopher J. Weston

**Affiliations:** 1 Ministry of Industry, Tourism, Forest and Environment, Pokhara, Gandaki, Nepal; 2 Faculty of Science, School of Ecosystem and Forest Sciences, the University of Melbourne, VIC, Australia; 3 Faculty of Forestry and Wood Sciences, Czech University of Life Sciences, Prague, Czech Republic; 4 United Nations University, Tokyo, Japan

**Keywords:** Allometric equation, Carbon sequestration, Community forest, REDD^+^

## Abstract

On a global scale, about 15.5% of forests are administered through community-based forestry programs that offer the opportunity for enhanced carbon sequestration while maintaining the supply of more traditional goods and services such as cooking fuels, animal fodder and bedding. A challenge in community forest (CF) management is to realize their carbon value without compromising their role in the provision of these traditional goods and services. In this study of CF dominated by *Pinus roxburghii* in the Phalebas region of Nepal, the impacts of stand composition and geographic aspect on aboveground forest carbon is investigated as a means to optimize CF management for both traditional values and for emerging carbon market values. The aboveground carbon of mixed and monospecific stands of *Pinus roxburghii* was estimated using a combination of destructive sampling and species-specific allometric equations. On average, monospecific stands contained 106.2 Mg C ha^−1^ in aboveground tree biomass, significantly more than mixed stands at 73.1 Mg C ha^−1^ (*p* = 0.022). Similarly, stands growing on northern aspects (northeast 124.8 Mg C ha^−1^, northwest 100.9 Mg C ha^−1^) stored significantly more carbon (*p* = 0.002) than southern aspects (southeast 75.3 Mg C ha^−1^, southwest 57.6 Mg C ha^−1^), reflecting the more favorable growing conditions of northern aspects. These results suggest monospecific stands planted on northern aspects may be best suited for management to achieve carbon benefits, whilst mixed-species stands on southern aspects may be better suited for biodiversity conservation and supporting livelihoods. To maintain and increase carbon value, community forestry may need to implement nutrient return practices to limit the impact of sustained nutrient removals on stand productivity.

## Introduction

Community forests (CFs) are widespread in developing economies and for decades have been a source of fuel, animal fodder and building materials for immediate use, as well as providing more strategic values such as biodiversity conservation, erosion risk mitigation and carbon sequestration (Luintel, Bluffstone & Scheller, 2018a; Persha, Agrawal & Chhatre, 2011). On a global scale, about 15.5% of forests are administered through community-based forestry programs (Rights and Resources Initiative, 2014). At a national scale, economies with extensive CFs will benefit from forest knowledge that enables the benefits of schemes such as reducing emission from deforestation and forest degradation (REDD^+^) to be realized, while not compromising existing community needs from these forests. Globally forest ecosystems sequester 66.5 billion tonnes C in aboveground biomass (FAO, 2015), with Nepal’s forests accounting for about 485 million tonnes C in aboveground biomass (FAO, 2015). Sustainable forest management to enhance the carbon sequestered in CFs can assist in mitigating and stabilizing elevated concentrations of atmospheric carbon dioxide (CO_2_) (Bhattarai et al., 2012; Shrestha et al., 2013).

Nepal’s Community Forestry Program (CFP) is one of the world’s most successful examples, involving local people in forest conservation and the wise management of forest resources for poverty reduction (Springate-Baginski et al., 2003; Sunam, Paudel & Paudel, 2013). The CFP was introduced in the late 1970s building on from the National Forestry Plan of 1976, and affirmed in the Master Plan for the Forestry Sector of Nepal 1989 and the Forest Act of 1993 (Dahal & Chapagain, 2008; Ojha, Persha & Chhatre, 2009). In Nepal, community-managed reforestation with *Pinus roxburghii* for soil conservation in the mid-hills region (Jackson, 1994) now accounts for almost nine percent of the total forest area, making *P. roxburghii* the third major species of Nepalese forests (DFRS, 2015). More recently the opportunity for management of *P. roxburghii* forests for enhancement of carbon stocks and as a sink of greenhouse gases (GHGs) has emerged as a potential benefit for local communities participating in carbon trading under REDD^+^ agreements (Shrestha et al., 2013; Pandey, Cockfield & Maraseni, 2016). However, the potential benefit to communities from carbon trading requires quantification of forest carbon stocks by accepted methods, followed by monitoring, reporting and verification to demonstrate enhancement of forest carbon.

Previous studies of forest carbon stocks in the government-managed *P. roxburghii* forests of India and Pakistan (Nizami et al., 2009; Sharma et al., 2011; Pant & Tewari, 2013, 2014) do not reflect Nepalese conditions, including potential community management impacts on carbon stocks. For example, in Nepal’s CFs, users extract woody debris and dead standing trees for firewood, and collect forest floor litter for cattle bedding (Sunam & McCarthy, 2010). These actions are in addition to routine silvicultural operations such as weeding, thinning, pruning and selective felling. A few Nepalese studies, such as Shrestha et al. (2013) estimated stem carbon of *P. roxburghii* forests in the Dolkha district, while Pandey, Cockfield & Maraseni (2014a) estimated forest carbon according to dense and sparse canopy classes. Luintel, Bluffstone & Scheller (2018a) studied biodiversity conservation and carbon storage in Nepal CFs, focusing mainly on quantification of carbon according to broad physiographic features and crown cover, but did not assess aboveground carbon (AGC) on the basis of the species of interest (*Pinus roxburghii*). In addition to these studies, others (e.g. DFRS, 2015; Luintel, Scheller & Bluffstone, 2018b; Maraseni & Pandey, 2014; Pandey, Maraseni & Cockfield, 2014b) focused more on the broad-scale assessment of carbon encompassing multiple forest species.

The objective of this study is to address a knowledge gap in stand-level and species-specific CF carbon of Nepal, by quantifying and evaluating the AGC based on stand type and geographic aspect, in *P. roxburghii* dominated forests. Standard inventory methods coupled with allometric biomass equations for trees (Sharma & Pukkala, 1990) and saplings (Tamrakar, 2000) and destructive sampling of understory vegetation was applied to estimate AGC. Data were acquired from 24 sample plots in community-managed monospecific and mixed *P. roxburghii* forests located at contrasting geographical aspects in western Nepal. It is anticipated the study will benefit communities managing the local forest by supporting them to realize the benefits of schemes such as REDD^+^ and in providing a more informed basis for decision-making in multi-purpose CF management of *P. roxburghii* in the future.

## Materials and Methods

### Study area

**Figure 1:**
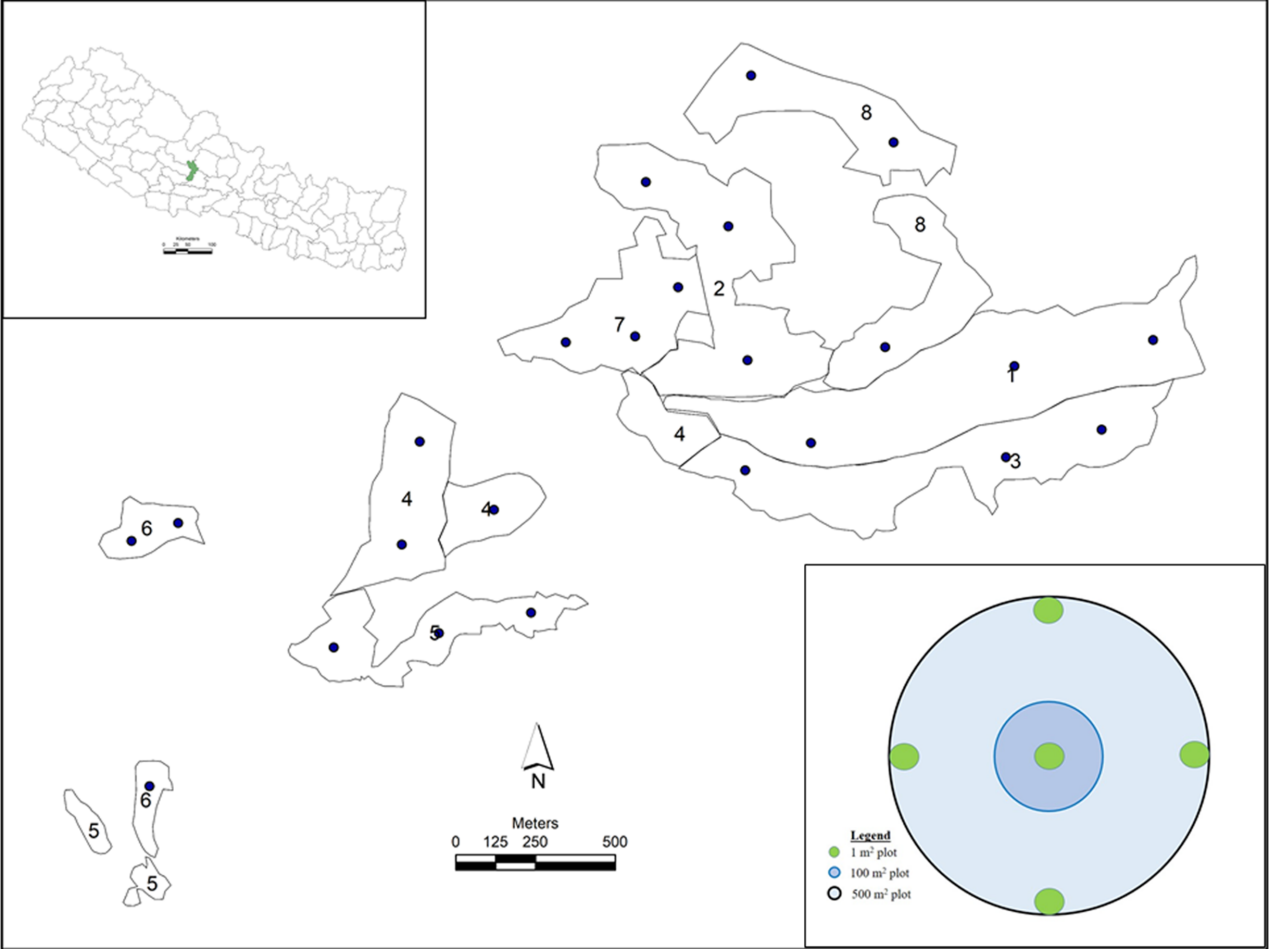
Study sites in CFs of Nepal. Study sites in CFs of Nepal, where 1) Monospecific, NE aspect; 2) Mixed, NE aspect; 3) Monospecific, SE aspect; 4) Mixed, SE aspect; 5) Monospecific, SW aspect; 6) Mixed, SW aspect; 7) Monospecific, NW aspect; and 8) Mixed, NW aspect. Black bullets in the study sites indicate sample plots. Concentric circles of three different sizes depict sample design of a plot. (Photo credit: Shiva Pariyar).

Community forests dominated by *P. roxburghii* in the Phalebas municipality of the Parbat district in western Nepal were selected for the study (Fig. 1). The study area lies between 28° 09′ 35″ to 28° 12′ 02″ North and 83° 39′ 07″ to 83° 42′ 37″ East over 800 m to 1,500 m above mean sea level. The study area has a sub-tropical climate with a minimum temperature of 2 °C in December–January and a maximum temperature of 37 °C in July–August and an annual precipitation of 2,500 mm, much of which falls from June until mid-September (DCC, 2017). The study area landform has moderate to precipitous slopes underlain by granite and gneiss rock types which form coarse to medium-textured, acidic and shallow soils. Accessible CFs with active forest management including weeding, cleaning, thinning, pruning, salvage cutting and enrichment planting were selected based on detailed information obtained from the constitutions and operational plans held at the District Forest Office (DFO) Parbat, and by the District Coordination Committee (DCC) Parbat (DCC, 2017; DFO, 2017). Sampling plots were established across eleven CFs of various sizes and consisting of both monospecific and mixed stands of *P. roxburghii* where the other species include *Schima wallichii* (DC.) Korth., *Shorea robusta* Roth, *Alnus nepalensis* D. Don, *Diospyrous malabarica* (Desr.) Kostel., *Myrica esculenta* Buch.-Ham. ex D. Don and *Castanopsis indica* (Roxb. ex Lindl.) A. DC. The CFs cover 161.63 ha and provide benefits to 6,160 people of 1,032 households (DFO, 2017). All forests of the study area have been conserved and managed by the local communities for the last four decades (since the late 1970s). Before that period, the stands were badly degraded due to overexploitation of resources.

### Sampling design

A total of twenty-four sample plots were established using a stratified random sampling design (Fig. 1). Study plots were randomly selected using ArcGIS 10.3 (Environmental Systems Research Institute Inc., Redlands, CA, USA), uploaded to a global positioning system for spatial navigation and ground-truthed. The shapefile of the study area was obtained from DCC and DFO Parbat.

Pure stands of *P. roxburghii* were classified as ‘monospecific’ and stands of *P. roxburghii* associated with other species were classified as ‘mixed’. Based on stand geographical location, forests were further classified into four aspects: Northeast (NE), Southeast (SE), Southwest (SW) and Northwest (NW), resulting in three sample plots for each sub-class of stand type and aspect, giving a total of 24 plots (2 stand types x 4 aspects x 3 replicates, Fig. 1, Table S1).

This study was focused on assessing AGC pools, while belowground carbon pools (tree roots and soil organic matter (SOM)) were not sampled due to limited resources. Circular sample plots of 0.05 ha were established to sample all aboveground vegetation according to stem diameter classes. Vegetation such as shrubs, herbs and grasses with a diameter at breast height (DBH, 1.3 m) of less than one cm were destructively sampled from five 1 m^2^ quadrats located at the center and at four opposite locations of a plot (Fig. 1). The ground layer in monospecific *P. roxburghii* forest was dominated by ferns and grasses with no tree seedlings regenerating. In mixed stands, regeneration of *Schima-Castanopsis* species was sparse with no regeneration of *P. roxburghii*. Samples of *Schima-Castanopsis* understory were taken from five nested plots, clipped, weighed and mixed together in-situ. A subsample of approximately 100 g from this mixture was taken to the laboratory and oven dried at 70 °C for 96 hours prior to re-weighing. The mass of understory vegetation was estimated on an oven dry basis. Trees with DBH above one cm but less than five cm (5 cm > DBH ≥ 1 cm; referred to as saplings) were measured from a 0.01 ha (5.64 m) subplot, while trees with DBH ≥ 5 cm were measured within a circular plot of 0.05 ha (12.61 m) (Fig. 1). All trees were identified to species level and measured for DBH (diameter tape); trees with DBH ≥ 5 cm were measured for height to the top of the canopy (Sunto-clinometer, linear tape) and for crown density (densiometer).

**Figure 2:**
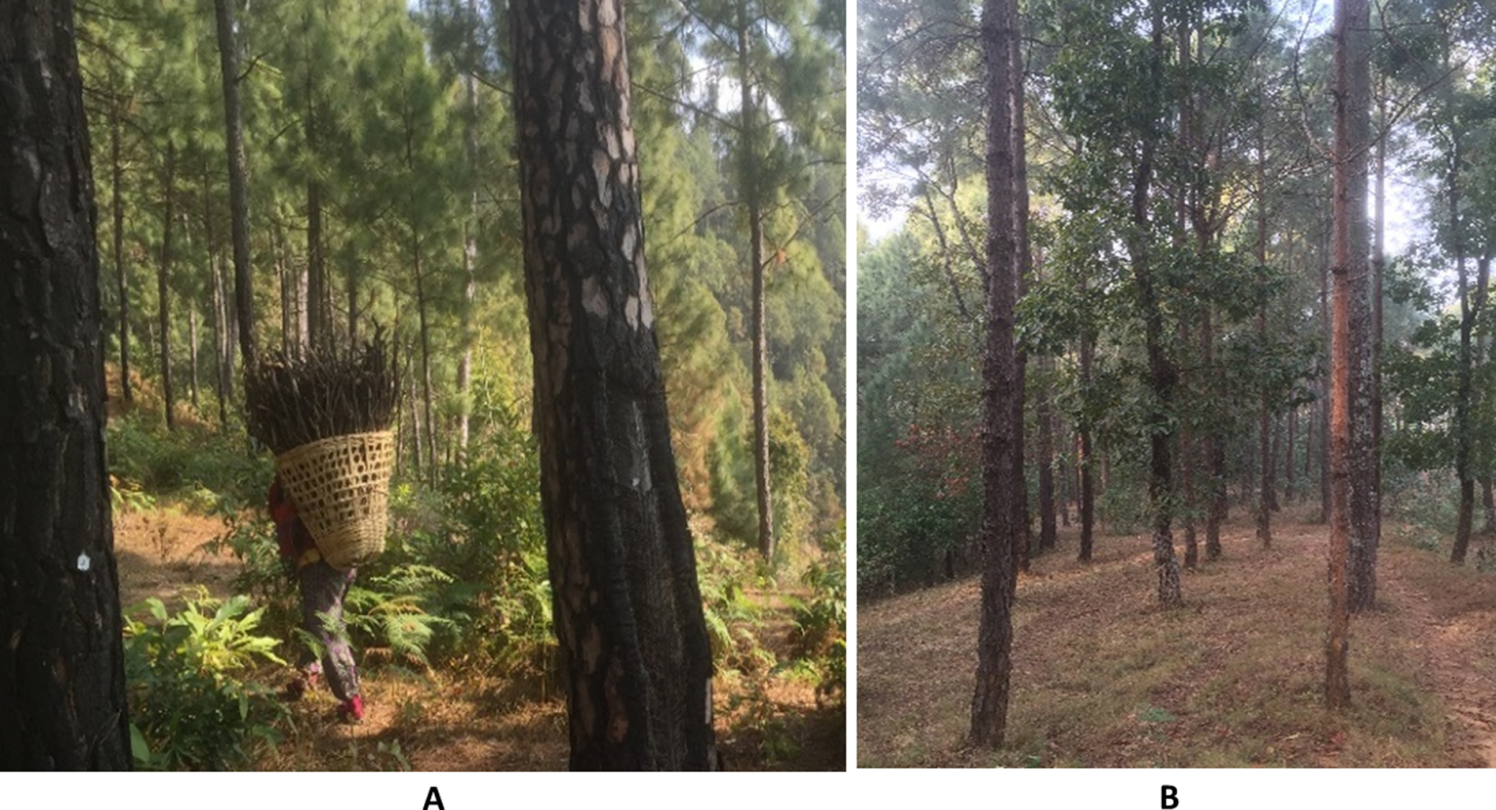
Photos of the community forest study area showing (A) local people collecting fuelwood and fallen needles for domestic use and (B) the resulting openness of the forest ground layer. Photo credit: Shiva Pariyar.

Deadwood (standing dead trees, stumps, coarse woody debris), and litter were not measured due to almost absence of these carbon pools in Nepal’s community-managed forests. CF users collect litter for cattle beds and deadwood for fuelwood, thus not allowing these pools to accumulate (Sunam & McCarthy, 2010) (Fig. 2). There is no specific study addressing the impact of litter and deadwood removal in our study sites. However, operational forest management plans (plans prepared by forest users in support of district forest officials) of the study area have clearly mentioned the provision of the amount of litter and deadwood to be collected.

### Estimating aboveground carbon

Aboveground tree biomass (DBH ≥ 5 cm) was calculated as sum of stem, branch and foliage biomass. Stem biomass was calculated from stem volume multiplied by species wood density (Jackson, 1994). Stem volume was calculated from the stem volume equation (Eq. (1)), (Sharma & Pukkala, 1990). Branch and foliage biomasses were computed from the stem biomass using species-specific branch to stem biomass and foliage to stem biomass ratios taken from the Master plan for the forestry sector of Nepal (MFSC, 1989), (Table S2)

(1) $${\rm{ln }}\left( {\rm{V}} \right) = {\rm{a}} + {\rm{bln}}\,\left( {{\rm{DBH}}} \right) + {\rm{cln}}\left( {\rm{H}} \right)$$

where: V, over bark stem volume (dm^3^); DBH, Diameter at breast height (cm); H, Total tree height (m); and a, b, c are species-specific parameters (Table S3).

Aboveground sapling biomass (5 cm > DBH ≥ 1 cm) was extracted from biomass tables for community-managed forests (Tamrakar, 2000). Total sapling biomass included leaf, branch and stem compartments. Understory biomass (UB) was calculated using destructive sampling (Eq. (2)).

(2) $$\eqalign{{\rm{UB}} = {{{\hbox{Weight of freshly felled sample in a plot (kg)}}} \over {{\hbox{Area of a plot (}}{{\rm{m}}^{\rm{2}}}{\rm{)}}}} \cr\quad* {{{\hbox{Dry weight of subsample (kg)}}} \over {{\hbox{Fresh weight of subsample (kg)}}}} * 10,000}$$

Aboveground tree biomass of *P. roxburghii* and *Schima wallichii* species was converted to carbon using species specific carbon content (Negi, Manhas & Chauhan, 2003). Specifically, for *P. roxburghii* wood carbon fraction was 0.4632 and leaves of 0.4346; for *Schima wallichii* wood carbon fraction was 0.4505 and leaves was 0.4352. For other species, saplings and understory vegetation, a default carbon fraction of 0.47 was applied (IPCC, 2006).

### Stastistical analysis

Student’s t-test was used to assess the significance of stand types (mono/mixed) and a one-way analysis of variance was used to assess the significance of aspects (NE, NW, SE, SW) on forest carbon stock (Zar, 2010) by using Minitab 18 (Minitab Inc. State College, State College, PA, USA). Data distribution was tested (frequency distribution) by each variable of interest for each stand type and aspect for the normality of distribution. The distribution pattern of variable of interest (DBH, Height) by stand types and aspects seemed approximately symmetric, balanced and regular. The student t-test showed that data distribution was normal (*p* = 0.0001, Table S4). Unless otherwise specified, 5% level of significance was used in all analyses in this study.

## Results

### Community forest stand composition

**Figure 3:**
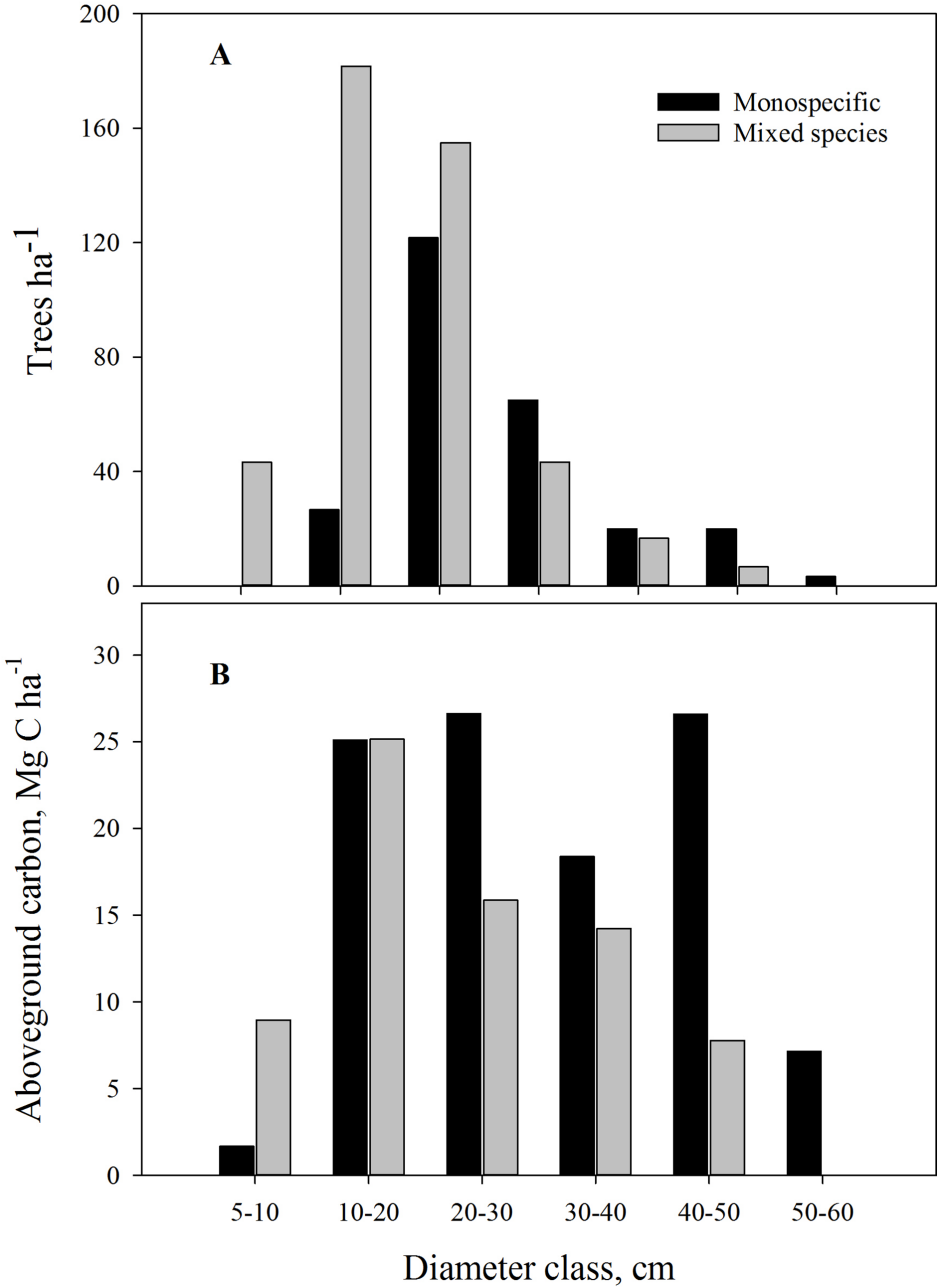
(A) Diameter class distribution in monospecific and mixed stands; (B) Average aboveground carbon by diameter class in the community forests of Parbat district, Nepal.

*Pinus roxburghii* is the dominant species (80%) at the study sites, followed by *Schima wallichii* (19%), *Myrica esculenta* (0.7%) and *Castanopsis spp.* (0.5%) (Table 1). Stand type and aspect were significant factors affecting tree DBH and height, with trees in monospecific stands of larger DBH and height compared to mixed stands. Stands facing north had 28% greater diameter and were 19% taller compared with south facing stands (Table 1). Mixed stands were dominated by trees with DBH up to 30 cm, and the monospecific stands contained more trees of larger diameter (Fig. 3A). Both mono and mixed stands had a similar number of sapling trees (Table 1).

**Table 1:** Community forest stand parameters according to stand type (monospecific or mixed species, *n* = 12) and site aspect (north east—NE, south east—SE, north west—NW, south west—SW, *n* = 6).

Parameters	All stands	Stand type		Stand aspect			
		Mono	Mixed	NE	SE	NW	SW
Trees (DBH ≥ 5 cm)							
*P. roxburghii* (%)	80.1	100	68.7	64.8	89.8	78.8	88.8
*S. wallichii* (%)	18.7		29.5	32.8	21.2	10.2	9.3
*M. esculenta* (%)	0.7		1.1	2.5			
*C. indica* (%)	0.5		0.7				1.9
DBH (cm)	28.5 ± 2.1	34.9 ± 3.1	22.1 ± 1.1	31.2 ± 6.5	25.1 ± 2.2	32.9 ± 4.6	24.8 ± 1.6
Height (m)	20.3 ± 1.3	24.3 ± 1.8	16.3 ± 0.8	22.2 ± 3.7	20.0 ± 1.3	21.9 ± 3.0	17.2 ± 1.6
Density (stems ha^–1^)	352 ± 36	257 ± 37	447 ± 49	407 ± 94	360 ± 59	283 ± 54	357 ± 83
Saplings (5 cm > DBH ≥1 cm)							
DBH (cm)	3.8 ± 0.1	3.9 ± 0.1	3.7 ± 0.1	3.5 ± 0.2	4.1 ± 0.1	3.9 ± 0.1	3.8 ± 0.1
Density (stems ha^–1^)	479 ± 37	458 ± 66	500 ± 37	333 ± 88	483 ± 48	467 ± 33	633 ± 72

### Community forest aboveground carbon

**Figure 4:**
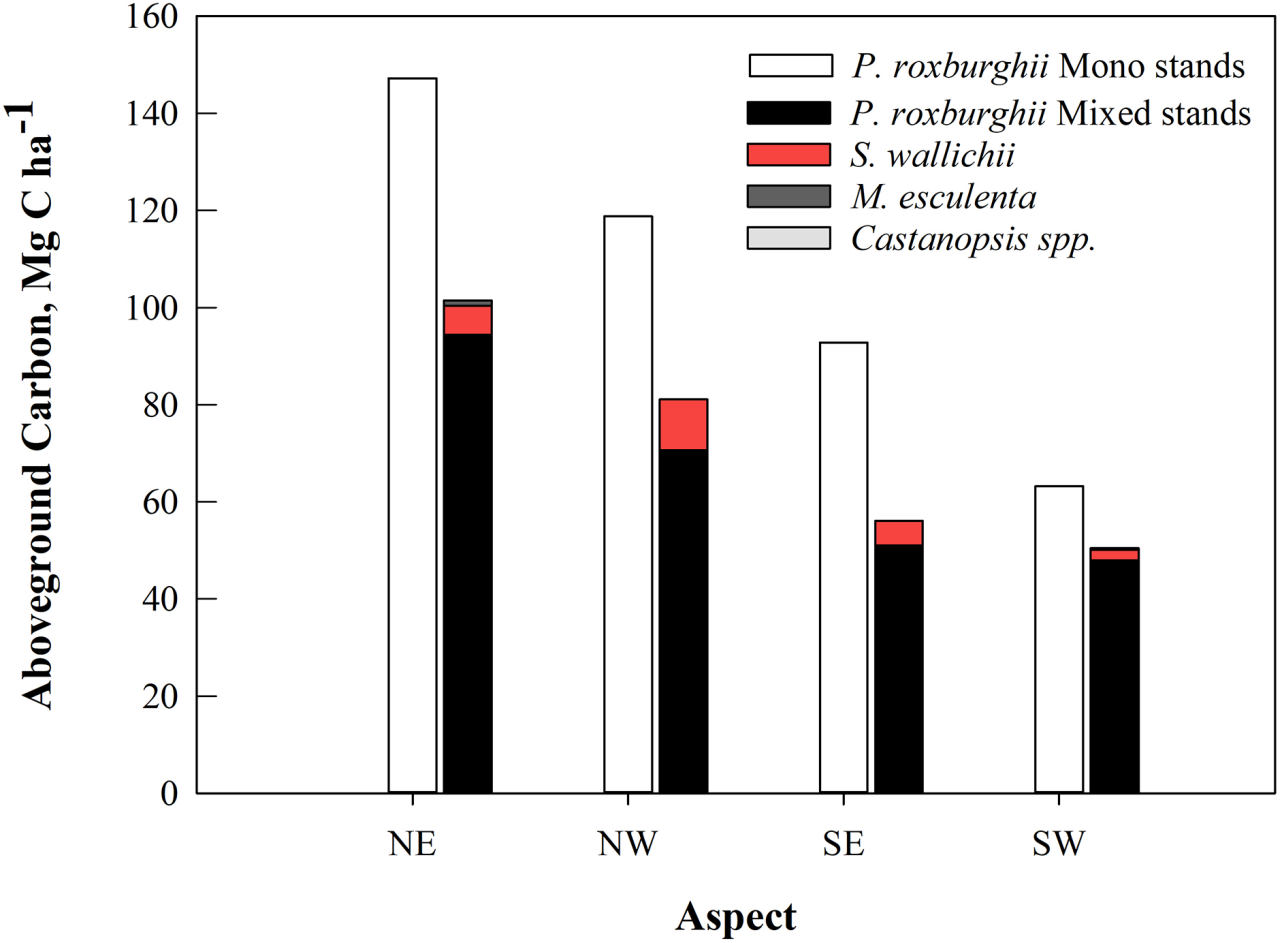
The average aboveground carbon (Mg C ha^−1^) by tree species in mixed and monospecific forest stands (*n* = 24) in the 11 community forests of Parbat district, Nepal.

Monospecific stands of *P. roxburghii* stored significantly more carbon than mixed-species stands, with most carbon in trees with DBH > 50 cm (Fig. 3B); these larger diameter trees were a minor component in mixed-species stands. Total standing AGC of tree species of the study area varied with species. *P. roxburghii* species dominated both mixed and monospecific stands and contained significantly greater carbon than other species (*p* = 0.022, Fig. 4). In mixed stands, *Schima wallichii* stored 5.9 Mg C ha^−1^, followed by *M. esculenta* with 0.25 Mg C ha^−1^ and *Castanopsis spp.* stored a negligible amount of AGC with 0.09 Mg C ha^−1^ (Fig. 4). Similarly, stands on north-facing slopes stored significantly greater amount of AGC (*p* = 0.002) than stands on south-facing slopes, following a decreasing trend from NE (124.8 Mg C ha^−1^) > NW (100.9 Mg C ha^−1^) > SE (75.3 Mg C ha^−1^) > SW (57.62 Mg C ha^−1^) (Table 2). The carbon stock of the whole forest averaged 89.65 Mg C ha^−1^ contributed 99.3% (88.98 Mg C ha^−1^) by trees, 0.5% (0.46 Mg C ha^−1^) by sapling and 0.2% (0.21 Mg C ha^−1^) by understory vegetation.

**Table 2:** Aboveground tree biomass (Mg C ha^−1^) of *P. roxburghii* forest by aspect and forest management.

Reference	Biomass by aspect				Biomass average	Forest management
	NE	NW	SE	SW		
This study	124.8 ± 16.3	100.9 ± 11.9	75.3 ± 8.9	57.6 ± 7.8	89.65 ± 7.4	Community forests
(Sharma et al., 2011)	165.2	106.4	108.0	61.7	110.33	Reserved forests
(Bhattarai et al., 2012)					91.86	Community forests
(Shrestha et al., 2013)					32.568	Community forests
(Pandey, Maraseni & Cockfield, 2014b)					∼79	Community forests
(Luintel, Scheller & Bluffstone, 2018b)					98.3	Community forests

## Discussion

The AGC in CFs were similar to other studies reported in the literature (Aryal et al., 2018; Bhattarai et al., 2012; DFRS, 2015; Luintel, Scheller & Bluffstone, 2018b; Pandey, Maraseni & Cockfield, 2014b) and data presented in Table 2. Consistent with several recent studies of *P. roxburghii* forest productivity, forest stands of northern aspects stored more carbon (Table 2). These results are not surprising and reflect that, compared with southern slopes, the northern aspects (in the northern hemisphere) have greater moisture availability than southern aspects (Jackson, 1994), hence providing more water for growth and development of trees. However, the carbon storage of these *P. roxburghii* community-managed forests is low compared to the government-managed *P. roxburghii* forests or reserved forests of India (Table 2). Apart from site productivity differences, the reason for this could be a younger age-class structure of the CFs, or it may result from human-induced over-thinning earlier in stand development.

The lower productivity of CFs may also be a consequence of nutrient decline due to continuing removals of forest floor litter, small branches and wood (Fig. 2) relative to protected forests stands. In particular, the sustained removal of litter (as well as deadwood) could reduce forest productivity through decreased rates of nutrient cycling through the forest floor, as litterfall is the major cycle of nutrient return from the forest canopy to the soil for root uptake. Around 70% of the annual uptake of macro-elements is returned to soil via litterfall in temperate forests (Duvigneaud & De Smet, 1973; Chaturvedi & Singh, 1987). It is likely that CF practices reduce forest floor mass to 1 Mg C ha^−1^ (Aryal et al., 2018; Bhattarai et al., 2012) compared with protected *P. roxburghii* forests with around 6 Mg C ha^−1^ (Chaturvedi & Singh, 1987). Supporting this are reports of soil fertility recovery on sites protected from biomass removal for at least 15 years relative to sites that have come under protection in the past 7 years (Schmidt, Schreier & Shah, 1993). In this study, litter and deadwood have been removed from mid-December to April for almost 25–40 years. This rate of biomass removal is likely to reduce nutrient availability in these shallow and steep granitic soils, where nutrients returned from aboveground through litterfall are an important source of annual nutrient requirement. To maintain and increase forest productivity and ensure sites are not depleted of SOM and nutrients over long periods of CF management, further investigation of the potential for nutrient decline is warranted. For example, the sustained annual removal of 30% of nutrients returned to the forest floor in annual litterfall (circa 8 Mg ha^−1^) (Chaturvedi & Singh, 1987) sums to about 24 Mg C ha^−1^, 1.2 Mg N ha^−1^ and 0.12 Mg P ha^−1^ over 10 years (assuming litter organic matter ratios are: C = 50%; C:N = 20; C:P = 200).

### Optimizing forest management to integrate carbon values with traditional CF values

The results of this study provide an inventory basis to develop policy that optimizes trade-offs between removed products such as fuelwood and litter, and the relatively new value of standing carbon stock in Nepal’s CFs. The broader range of species and tree sizes of mixed stands provides a wider range of products for community use (Smith & Scherr, 2003; Chhatre & Agrawal, 2009) and biodiversity. This is in contrast to monospecific stands that are likely more well-suited for management as a carbon stock compared with mixed stands (Shrestha et al., 2013). The results presented here show that *P. roxburghii* is well-suited for management as a carbon stock in CFs; furthermore it is a fast-growing and productive pioneer species that can reach over 50 m height and up to 1 m in DBH (Jackson, 1994). However, while it can be suggested that the monospecific stands are a preferred option for carbon-oriented forest management, mixed stands contribute more to local people’s livelihoods and promote biodiversity (Smith & Scherr, 2003; Chhatre & Agrawal, 2009). This study was focused on the carbon aspect of CF and did not consider biodiversity parameters such as species richness or plant species diversity. The observed regeneration of dominant overstory species in the mixed stands contrasted with no regeneration in monospecific stands, indicating the ongoing biodiversity value of mixed stands. Moreover, a study by Maraseni & Pandey (2014) observed that soils under mixed-species forests have higher amounts of SOM than that of single species dominated forests, indicating the importance of quantifying SOM in determining carbon storage benefits of CFs. Several studies indicated a positive effect of CF management for biodiversity compared to non-CF (Bowler et al., 2012; Luintel, Bluffstone & Scheller, 2018a). Carbon focused forestry practices also contravene the social and environmental safeguard principle (UNFCCC, 2011). Only forests with diverse species can provide multiple livelihood benefits to rural communities (Arnold et al., 2011) and such forests are likely to play an important role in the resilience of local rural communities in developing countries such as Nepal.

The management of forests to increase sequestered carbon is a proven efficient and economical method for off-setting GHG emissions from other areas of the economy and constraining CO_2_ accumulation in the atmosphere (Pearson et al., 2014; Gren & Aklilu, 2016). The relatively high carbon storage in the CFs studied here indicates a potential to increase carbon storage in Nepal’s CFs with enhanced management (Pandey, Cockfield & Maraseni, 2014a; Luintel, Scheller & Bluffstone, 2018b). Increase in carbon stock can also bring benefits under the REDD^+^ scheme as demonstrated in a study by Maraseni et al. (2014) where incentives of REDD^+^ payments led to a reduction in the extraction of litter, grass and fodder materials from CFs, increasing both their productivity and sustainability.

## Conclusion

This study has estimated aboveground forest carbon stock of community-managed *P. roxburghii* forests of the Parbat district of Western Nepal. In *P. roxburghii* dominated forest, trees account for over 99% of total aboveground forest carbon, indicating that carbon measurements can be focused only on trees in these CFs. This will reduce the time and cost involved in field measurements and simplify the task of planning, monitoring, reporting and verification under the REDD^+^ framework. This study has shown that AGC stock in community-managed forests varies with both stand type and geographical aspect. Monospecific stands contained more biomass carbon than the mixed stands, suggesting that former are a preferred option for carbon-oriented forest management. However, mixed stands contribute more to local people’s livelihoods and promote biodiversity. For optimum outcomes in terms of accumulating carbon, conserving biodiversity, and supporting local people’s needs, forest management efforts can be directed to promoting monospecific stands on northern aspects and mixed stands on southern aspects. This can be regarded as an important policy insight and will be useful for policymakers in multi-purpose forest management. On a cautionary note, the potential for community forestry to decrease forest productivity due to removal of forest floor materials warrants careful investigation in well-replicated field trials to test the impact of sustained nutrient removals on stand productivity.

## Supplemental Information

10.7717/peerj.6494/supp-1 Supplemental Information 1 Plot level information of the study sites.

10.7717/peerj.6494/supp-2 Supplemental Information 2 Oven dry wood densities and biomass ratios of tree species of the study area.

10.7717/peerj.6494/supp-3 Supplemental Information 3 Species-specific estimated parameters.

10.7717/peerj.6494/supp-4 Supplemental Information 4 t statistic and *p*-values of DBH and Height after examination of normality of data distribution of the stands.

10.7717/peerj.6494/supp-5 Supplemental Information 5 Inventoried and analyzed data.

## Additional Information and Declarations

### Data Availability

The following information was supplied regarding data availability: Pariyar, Shiva; Volkova, Liubov; Sharma, Ram P.; Sunam, Ramesh; J. Weston, Christopher (2019): Inventoried and Analyzed Data.xls. figshare. Dataset. https://doi.org/10.6084/m9.figshare.7660229.v1.
