# Supplementary material for: Aboveground carbon of community-managed Chirpine (Pinus roxburghii Sarg.) forests of Nepal based on stand types and geographic aspects
Source: PeerJ. 2019 Mar 8;7:e6494. doi: 10.7717/peerj.6494 (PMC6410687; doi:10.7717/peerj.6494)
Supplement: Supplemental Information 1 [file peerj-07-6494-s001.docx]

| **Plot #** | **Community Forest name** | **Stand type** | **Altitude, m** | **Aspect** |
| --- | --- | --- | --- | --- |
| 1 | Koldanda | Mixed | < 1000 | NE |
| 2 | Bhumethan | Mixed | > 1300 | NE |
| 3 | Thulo Pakho | Mixed | 1000 - 1300 | NE |
| 4 | Koldanda | Mixed | < 1000 | NW |
| 5 | Laligurans | Mixed | > 1300 | NW |
| 6 | Hariyali | Mixed | 1000 - 1300 | NW |
| 7 | Koldanda | Mixed | < 1000 | SE |
| 8 | Shivashankar | Mixed | > 1300 | SE |
| 9 | Thulo Pakho | Mixed | 1000 - 1300 | SE |
| 10 | Bayardanda | Mixed | < 1000 | SW |
| 11 | Bhumethan | Mixed | > 1300 | SW |
| 12 | Gahate Armana | Mixed | 1000 - 1300 | SW |
| 13 | Jayashankar | Monospecific | < 1000 | NE |
| 14 | Jayashankar | Monospecific | > 1300 | NE |
| 15 | Bayardanda | Monospecific | 1000 - 1300 | NE |
| 16 | Jayashankar | Monospecific | < 1000 | NW |
| 17 | Nabha Pratibha | Monospecific | > 1300 | NW |
| 18 | Nabha Pratibha | Monospecific | 1000 - 1300 | NW |
| 19 | Lamakhora | Monospecific | < 1000 | SE |
| 20 | Gahate Armana | Monospecific | > 1300 | SE |
| 21 | Gahate Armana | Monospecific | 1000 - 1300 | SE |
| 22 | Koldanda | Monospecific | < 1000 | SW |
| 23 | Shivashankar | Monospecific | > 1300 | SW |
| 24 | Shivashankar | Monospecific | 1000 - 1300 | SW |
