# Supplementary material for: Aboveground carbon of community-managed Chirpine (Pinus roxburghii Sarg.) forests of Nepal based on stand types and geographic aspects
Source: PeerJ. 2019 Mar 8;7:e6494. doi: 10.7717/peerj.6494 (PMC6410687; doi:10.7717/peerj.6494)
Supplement: Supplemental Information 2 [file peerj-07-6494-s002.docx]

| **Species** | **Density (kg m^-3^)** | **Branch to stem biomass** | | | **Foliage to stem biomass** | | |
| --- | --- | --- | --- | --- | --- | --- | --- |
|  |  | **Pole** | **Small timber** | **Large timber** | **Pole** | **Small timber** | **Large timber** |
| Chirpine (*P. roxburghii*) | 650 | 0.189 | 0.256 | 0.300 | 0.101 | 0.046 | 0.033 |
| Chilaune (*Schima wallichii*) | 690 | 0.520 | 0.186 | 0.168 | 0.064 | 0.035 | 0.033 |
| Katus (*Castanopsis spp.*) | 740 | 0.398 | 0.915 | 1.496 | 0.053 | 0.048 | 0.042 |
| Hadekafal (*Myrica esculenta*) | 750 | 0.524 | 0.590 | 0.605 | 0.170 | 0.160 | 0.155 |
