# Supplementary material for: Aboveground carbon of community-managed Chirpine (Pinus roxburghii Sarg.) forests of Nepal based on stand types and geographic aspects
Source: PeerJ. 2019 Mar 8;7:e6494. doi: 10.7717/peerj.6494 (PMC6410687; doi:10.7717/peerj.6494)
Supplement: Supplemental Information 3 [file peerj-07-6494-s003.docx]

| **Species** | **Species - specific parameters** | | | |
| --- | --- | --- | --- | --- |
|  | **a** | **b** | **c** | **R^2^** |
| Chirpine (*P. roxburghii*) | -2.9770 | 1.9235 | 1.0019 | 99.2 |
| Chilaune (*Schima wallichii*) | -2.7385 | 1.8155 | 1.0072 | 98.3 |
| Miscellaneous hill species | -2.3204 | 1.8507 | 0.8223 | 97.7 |
