# Supplementary material for: Aboveground carbon of community-managed Chirpine (Pinus roxburghii Sarg.) forests of Nepal based on stand types and geographic aspects
Source: PeerJ. 2019 Mar 8;7:e6494. doi: 10.7717/peerj.6494 (PMC6410687; doi:10.7717/peerj.6494)
Supplement: Supplemental Information 4 [file peerj-07-6494-s004.docx]

|  | **DBH** | | **Height** | |
| --- | --- | --- | --- | --- |
|  | **t statistic** | ***p* - value** | **t statistic** | ***p* - value** |
| Monospecific | 36.32 | 0.0001 | 46.15 | 0.0001 |
| Mixed | 36.21 | 0.0001 | 43.25 | 0.0001 |
| Northeast | 18.45 | 0.0001 | 23.41 | 0.0001 |
| Northwest | 22.42 | 0.0001 | 25.0 | 0.0001 |
| Southeast | 30.78 | 0.0001 | 42.91 | 0.0001 |
| Southwest | 33.43 | 0.0001 | 32.59 | 0.0001 |
| Overall stand | 46.1 | 0.0001 | 55.05 | 0.0001 |
